# The Potential of Cardiac Telerehabilitation as Delivery Rehabilitation Care Model in Heart Failure during COVID-19 and Transmissible Disease Outbreak: A Systematic Scoping Review of the Latest RCTs

**DOI:** 10.3390/medicina58101321

**Published:** 2022-09-21

**Authors:** Sidik Maulana, Yanny Trisyani, Ristina Mirwanti, Shakira Amirah, Kelvin Kohar, Aprilia Inggritika Priyatmoko Putri, Evi Novianti

**Affiliations:** 1 Professional Nursing Program, Faculty of Nursing, Universitas Padjadjaran, Bandung 45363, Indonesia; 2 Department of Critical Care and Emergency Nursing, Faculty of Nursing, Universitas Padjadjaran, Bandung 45363, Indonesia; 3 Undergraduate Medical Education, Undergraduate Faculty of Medicine, Universitas Indonesia, Depok 16424, Indonesia

**Keywords:** cardiac rehabilitation, heart failure, telerehabilitation

## Abstract

*Background and objective:* Patients with heart failure are a high-risk group who may have a higher mortality rate if infected during the COVID-19 pandemic. The problem of a patient’s non-adherence to cardiac rehabilitation programs is still a challenge, resulting in disappointing long-term benefits of cardiac rehabilitation. Telehealth, including telerehabilitation, has grown in popularity to improve access to quality healthcare. It is more valuable and safer compared to usual rehabilitation care, especially during the current COVID-19 pandemic, to cut down unnecessary hospital visits and reduce the risk of cluster infections. This study aims to identify the efficacy of relevant randomized control trials (RCTs) using telerehabilitation in managing heart failure. The model, delivery care, safety, and efficacy were assessed. *Material and* *Methods*: This study was conducted according to the Preferred Reporting Items for Systematic Reviews and Meta-analysis for Scoping Reviews (PRISMA-ScR). The authors included relevant records published in the last ten years from three databases: PubMed/MEDLINE, ProQuest, and EBSCO. Each included study was further assessed using Cochrane’s Risk of Bias (Rob 2) tool. *Results*: The telerehabilitation models consisted of cellphones, instant messaging, or online videoconferencing software. Some also included tool sets to monitor patients’ vital signs regularly or during exercise. Most patients adhered to and completed all provided programs. Cardiac telerehabilitation successfully improved patients’ physical fitness, quality of life, and mental health. No major adverse outcomes or significant complications were associated with the program. *Conclusion:* Cardiac telerehabilitation has the potential to deliver rehabilitation for heart failure patients, evidenced by its feasibility, efficacy, and safety. As a future perspective, this delivery care type can be applied throughout transmissible disease outbreaks or even globally.

## 1. Introduction

Patients with heart failure are a high-risk group who may have a higher mortality rate if infected during the COVID-19 pandemic [1]. Although the entire population of the world is susceptible to this virus, those who are older, male, severely obese, have preexisting comorbidities, or have underlying cardiovascular disease, including heart failure, have a poor prognosis [2]. While lockdown is a viable method of preventing viral spread, it restricts routine follow-up visits and precludes direct medical assessments necessary for detecting heart failure progression, optimizing treatment, and rehabilitation [3]. Additionally, lifestyle changes such as dietary changes, increased alcohol consumption, and decreased physical activity may result in heart failure decompensation during quarantine [3,4,5]. Beyond the pandemic, the problem of patients’ non-adherence to cardiac rehabilitation programs is still a challenge. Consistently, poor adherence and poor lifestyles often result in disappointing long-term benefits of cardiac rehabilitation.

Cardiac rehabilitation is a comprehensive intervention to prevent cardiovascular disease in the secondary prevention stage. It includes improving cardiovascular fitness through physical activity and other activities such as behavioral changes, risk factor modification, nutritional counseling, and psychosocial stimulation [6,7]. The goal of the cardiac rehabilitation program is to prevent the recurrence of cardiovascular disease, lower the risk of cardiovascular complications, and improve patients’ mental health and quality of life [8]. Throughout a transmissible outbreak, cardiac rehabilitation activities may suffer a devastating impact. Therefore, new strategies that can be used in addition to or in place of cardiac rehabilitation are needed now. These strategies could lead to greater participation and long-term therapy adherence and lessen the spread of the virus.

The current outbreak situation provides a suitable context within which to start a structured program in clinical practice, because COVID-19 can strongly affect patients, especially HF patients [9]. The program’s main advantages are that it helps to guide the care of patients in cardiac care, even primary care. Hence, the spread of the disease during cardiac rehabilitation to patients with heart failure is prevented, and unnecessary hospital visits are reduced, so as to reduce the risk of cluster infections [9,10]. Numerous studies have proven that telehealth can improve chronic disease management [11,12,13,14,15]. However, so far, only limited research has been published reviewing the model of telehealth or, specifically, the telerehabilitation model in the cardiac rehabilitation of heart failure [16]. At the same time, a previous study has reviewed the efficacy of the cardiac rehabilitation of heart failure but has not reviewed the model and efficacy regarding the New York Heart Association (NYHA) and left ventricle ejection fraction (LVEF) outcome [16].

In response to the new transmissible outbreak, assessing the model, delivery service, feasibility, and efficiency of telerehabilitation among the heart failure population is critical. This study’s findings may benefit nurses and other professionals, app developers, social groups, and scientific institutions. A scoping review of various telerehabilitation models can help developers to understand existing cardiac telerehabilitation programs’ shortcomings and help to develop new features and creative technology. The rapid spread of technology use also supports the use of telerehabilitation, leading to better accessibility for patients with HF. Nurses and other healthcare professionals can help patients to better manage their health by recommending the most efficient cardiac telerehabilitation model. In addition, this systematic scoping review related to the telerehabilitation model helps to provide a reference model that various countries can use according to their demographic conditions and resources. In an appropriate model, nursing care and multidisciplinary healthcare activities such as heart failure patient monitoring and rehabilitation can be performed online, saving time and money while reducing outbreak transmission, especially COVID-19. This is also supported by the fact that telerehabilitation for heart failure has not shown any side effects in patients with HF [17].

In this sense, this review aims to investigate the potential of telerehabilitation thoroughly, in terms of feasibility, safety, and efficacy aspects. This evaluation is expected to present an overview of the model and the potential of cardiac telerehabilitation as a rehabilitation care delivery model for heart failure throughout the COVID-19 and other transmissible disease outbreaks; it also may be of benefit to tackle cardiac rehabilitation adherence.

## 2. Materials and Methods

### 2.1. Study Design

The Preferred Reporting Items for Systematic Reviews and Meta-analysis for Scoping Reviews (PRISMA-ScR) [18] were used to conduct this study (see Supplementary File S1). The broad objective of this study was to investigate the telehealth model used for cardiac rehabilitation in heart failure patients, with a specific objective of examining its care delivery, safety, and efficacy.

### 2.2. Search Strategy

A literature search was performed through three databases, namely PubMed/MEDLINE, ProQuest, and EBSCO. Records that were published in the last ten years were included in this study. Search strategy and Boolean operators used on each scientific database are mentioned in Appendix A.

### 2.3. Eligibility Criteria

Throughout the process of developing this review, we applied inclusion criteria based on the PICOS framework and an amended review protocol developed by Komariah et al. (2021) [14].

P (Population): Heart faillure patients;
I (Intervention): Cardiac telerehabilitation;
C (Comparison): Not applicable;
O (Outcome): Delivery rehabilitation care model, safety, and efficacy;
S (Studies): Randomized controlled trial studies.

Other than the items listed above, publications with no access to the full text, studies written in a language other than English, and publications published before 2012 were excluded.

### 2.4. Data Extraction

Three authors carried out data extraction, and any discrepancies were later resolved by reaching a consensus among the group. The PRISMA protocol was used to extract information from the reviewed studies: identification of duplicates; screening of titles and abstracts; and availability of full texts are all aspects of the process [19]. The tabulation method was used to manually extract data from the study results. Author, study location, study design, nursing delivery care, model, and effectiveness were the extraction data of interest. The thematic qualitative method was used to analyze the data in this study.

### 2.5. Quality Assessment

The Risk of Bias (ROB) assessment was carried out following Cochrane’s Risk of Bias (Rob 2) tool [20]. Each study is evaluated for five quality parameters: randomization processes, deviations from intended interventions, missing outcome data, outcome measurement, and selection of reported results. Each domain is equipped with three to four signaling questions to help determine whether the final score represents a low, high, or unclear risk of bias in the data. The first three domains would be evaluated for concerns about the applicability of the research question in the context of the first three domains. The assessment was completed by both reviewers (SA and KK), with any discrepancies resolved by consensus and adjudication by a third reviewer after a consensus was reached (SM).

## 3. Results

### 3.1. Study Selection

**Figure 1:**
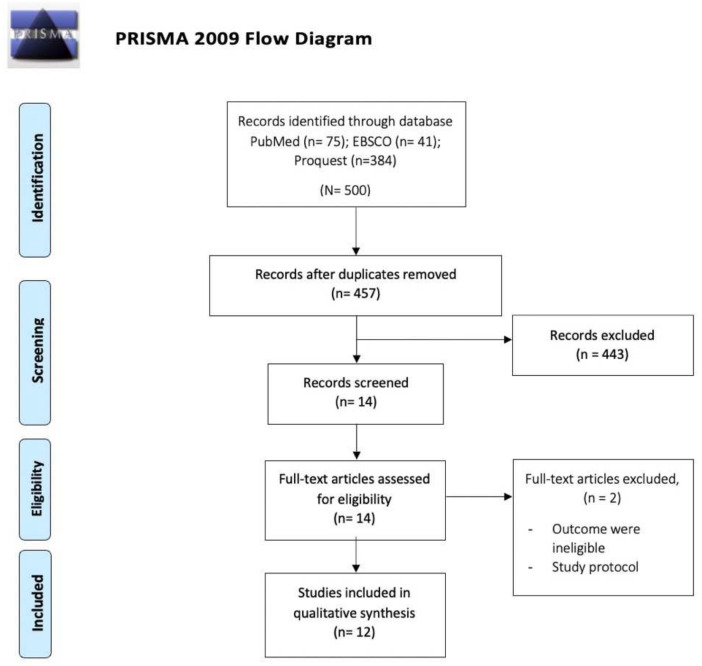
PRISMA flow diagram [21].

The flowchart in Figure 1 depicts the process of conducting a literature search. Through comprehensive searches, a total of 500 studies were identified. After eliminating 43 duplicates, the authors screened titles and abstracts, and 12 studies were retrieved for full-text evaluations. We excluded two studies because their protocol study and outcomes were ineligible. As a result, thirteen studies were included and subjected to quantitative analysis.

### 3.2. Characteristics of Included Studies

All included studies were randomized controlled trials (RCT) published between 2015 and 2021. Among the twelve included studies, eight studies were conducted in Poland, two in Australia, and one each in Italy and China [22,23,24,25,26,27,28,29,30,31,32]. Most participants were male, comprising heart failure patients (see Table 1).

### 3.3. Study Outcome

#### 3.3.1. Cardiac Telerehabilitation Model

We found numerous media to provide cardiac telerehabilitation for heart failure patients, such as mobile phones, instant messaging, and online videoconferencing software [22,23,29,30]. Table 2 shows the models developed for heart failure patients. Remote monitoring of patients via telecommunications technology and treatment centers in healthcare or cardiac centers was the main component of all existing models.

First, smartphones were used to assess and collect patient information related to symptoms or their current health, including fatigue, dyspnea, blood pressure, and body mass. The nurse used the smartphone to make phone calls to collect nutrition, lifestyle, and medication information. In addition, telephone interaction was utilized for mental healthcare. Patients were taught how to use all exercise training modalities, such as using the Borg scale and performing a telerehabilitation set. Dietary counselling, cholesterol management, smoking cessation, and psychosocial support were also provided with a smartphone [22]. Second, they still used part or all of the smartphone technology, with instant messaging media used for instant communication with nurses via text, audio, or video during exercise or rehabilitation at home [23]. Questions and responses were followed up with talks regarding the current situation and challenges. Referral services were also provided as needed [23]. Third, online videoconferencing software was used to conduct participants’ training and education using recorded audio files [30,33]. In addition to the above media, other modalities to support the diagnosis and monitoring of heart failure patients using objective data were designed to be used during rehabilitation at home, such as a one-lead or three-lead ECG. The ECG is designed with software to be automatically transmitted through smartphones and has an LED sensor that produces an alert when exercise needs to be stopped. In other modalities, the heart failure patient is given an oximeter and tensiometer [29]. Another model used in telerehabilitation for patients with heart failure is a hybrid model, where activities are carried out at home and in a cardiac center or health facility.

#### 3.3.2. Delivery Rehabilitation Care Service

The delivery services provided in the cardiac telerehabilitation model are the same as the usual rehabilitation care model, including current health assessment, and physical activity, dietary, and lifestyle evaluations. The only difference is that the service is remotely monitored through simple telecommunication media such as smartphones and additional telemonitoring devices using the software. By using this approach, patients can be monitored regularly within a short period of time. In addition, some models provide real-time monitoring, especially for patients’ vital signs during exercise.

#### 3.3.3. Feasibility, Safety, and Efficacy

Table 3 shows details of the cardiac telerehabilitation models’ feasibility, safety, and efficacy. Our study found that all existing telerehabilitation models were practical for patients seeking to complete cardiac rehabilitation at home, particularly during the necessary physical exercises. The telerehabilitation group had significantly higher attendance rates than the control group, who received usual rehabilitation care. Recent studies show that cardiac telerehabilitation is beneficial in enhancing exercise capacity, as demonstrated by improvements in the 6-min walking distance (MWD) scores [22,30,32] and improvements in cardiopulmonary exercise capacity [25]. Although only certain domains of quality of life were improved by cardiac rehabilitation interventions, telerehabilitation succeeded in significantly improving heart failure patients’ quality of life [22,24,26,27,32]. Moreover, functional status was improved in patients, most of whom were elderly, as evidenced by an increase in the Barthel score and a significant difference from the control group [22].

Concerning the safety of cardiac telerehabilitation, evaluation of cardiac arrhythmias and other clinical outcomes has been performed. During telerehabilitation, 320 (83%) individuals had a sinus rhythm, whereas 66 (66%) had chronic AF (17%). Premature ventricular and atrial beats were found in 76.4% and 27.7% of patients. The most common cardiac arrythmia was atrial fibrillation (six episodes in four patients) [26]. There was no difference in the mortality rate, where telerehabilitation had a mortality rate of 12.5% compared to the usual-care arm with a mortality rate of 12.4% (HR, 1.03 (95% CI, 0.70–1.51)). Hospitalization rates also did not differ, with HR 0.94 (95% CI, 0.79–1.13). In the outcome of the prognostic assessment, which was measured by peak oxygen, it was found that there was an increase in peak oxygen [26,31,32]. The peak oxygen consumption (VO2) of patients suffering from chronic HF, defined as the oxygen intake at the most significant degree of tolerated exertion, is a descriptive indicator with prognostic and decisional implications in patients suffering from chronic HF [35]. A study by Mancini et al. discovered that patients with a peak VO2 of less than 10 mL/kg per min had the worst prognosis of any group [35,36]. Related to the heart failure condition, telerehabilitation had a non-significant effect. The study showed that there was no significant worsening in the New York Heart Association (NHYA) class and no significant increase in left ventricular ejection fraction (LVEF) [23]. Moreover, telerehabilitation improves mental health in terms of mental health outcomes. Several studies have found that telerehabilitation significantly reduces levels of anxiety and depression [23,31].

#### 3.3.4. Quality of Included Studies

**Figure 2:**
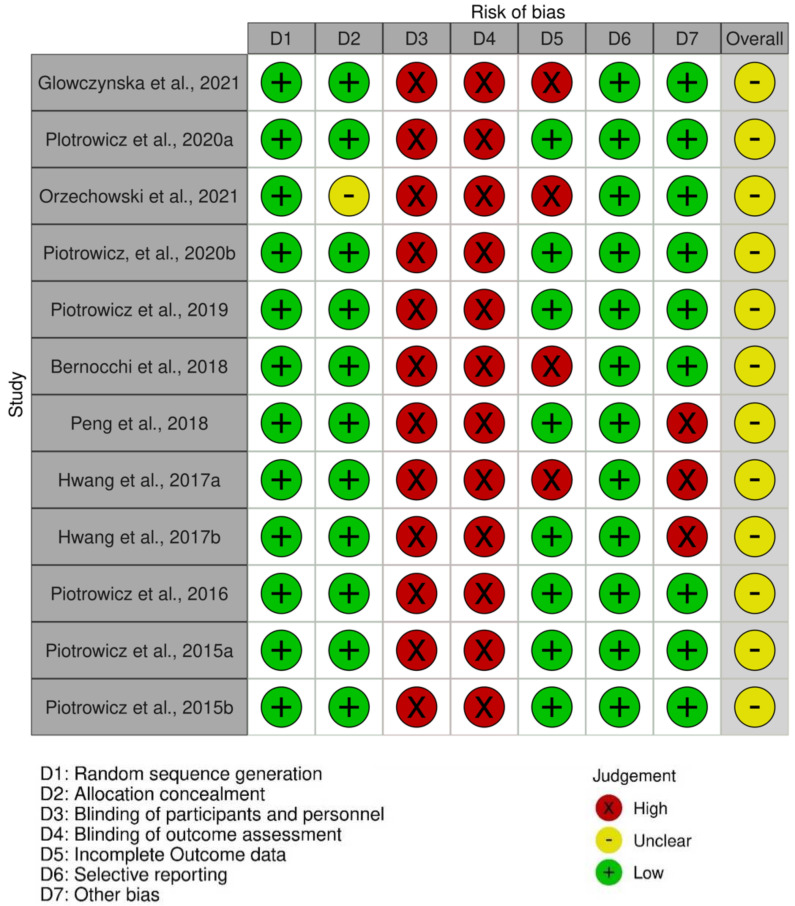
Traffic light plot of risk of bias.

**Figure 3:**
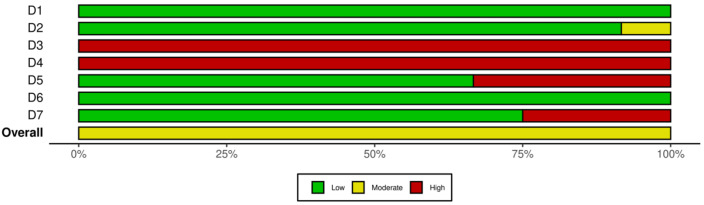
Summary of risk of bias.

A critical appraisal of the included studies found that the entire study carried a moderate risk of bias. Each assessment per study can be seen in the traffic light plot (see Figure 2) and the summary risk of bias in Figure 3. Several assessment components with low bias include random sequence generation and selective reporting components. High-risk assessment components are blinding participants and personnel, which are impossible to apply in the programs.

## 4. Discussion

This scoping review is the first review assessing the potential of cardiac telerehabilitation to be applied in transmissible outbreaks. The main findings of this review are as follows: (1) the media used in telerehabilitation models include smartphones, instant messaging, and online videoconferencing software; (2) cardiac telerehabilitation is feasible to use in heart failure patients; (3) telerehabilitation is effective in improving quality of life, peak oxygen consumption, exercise capacity, and mental health; (4) cardiac telerehabilitation is not inferior and is safe compared to the standard usual care.

The effectiveness of telerehabilitation was examined in a meta-analysis, ExTraMATCH. This study shows that cardiac telerehabilitation reduces mortality and improves cardiovascular prognosis [37]. Another meta-analysis of comparative reviews showed that patients had much better functional ability as measured by the 6-min walking distance, peak oxygen uptake (pVO2) values, and quality of life, and reported no adverse events during the program [16]. Batalik et al. (2021) [38] have demonstrated that cardiac telerehabilitation produces long-term improvements in pVO2, exercise performance, and overall health perception in coronary artery disease patients with low to moderate cardiovascular risk. This study is in line with our study findings [23]. In addition, another meta-analysis study showed that telerehabilitation effectively lowered blood pressure and reduced the length of hospital stays in patients with ischemic heart disease, hypertension, and heart failure [15]. Unfortunately, the implementation of cardiac telerehabilitation also raises unique concerns, such as training adherence. Therefore, the difference in the intensity and time of exercise is something that requires particular attention. Studies on this have been carried out, with the results indicating that the intensity of cardiac telerehabilitation compared to conventional cardiac telerehabilitation is similar [39] In terms of adherence, the study by Batalik et al. showed similar adherence between telehealth CR and conventional outpatient CR in CAD patients with low to moderate cardiovascular risk. This may be due to the loss of 4.8% of training data in the telehealth CR group [39].

Various telerehabilitation models have been developed for heart failure patients. All models show positive feasibility, safety, and efficacy to be adapted for rehabilitation care delivery during infectious disease outbreaks. All models use smartphones (instant messaging, telephone, online videoconferencing) for assessment, monitoring, education, and counseling, making them also applicable in developing countries. Another model consists of telerehabilitation sets, including one- or two-lead ECGs, oximeters, and automatic sphygmomanometers to optimize home care rehabilitation [21,29,30,33]. The problem of limited tool sets can be alleviated through hybrid methods. Based on a qualitative study, patients prefer the hybrid method, although they are satisfied with telerehabilitation overall [29]. The hybrid model is an alternative that can still fulfill patients’ social role to optimize their quality of life.

A meta-analysis reviewing the effects of exercise training in patients with chronic heart failure has demonstrated that exercise without a supervisor in heart failure patients might be dangerous. However, exercise training is also associated with a mortality reduction [37]. The appearance of telerehabilitation successfully addresses these problems, with the supervision of patients directly and indirectly during exercise training and daily activities. Therefore, patients can perform the exercise program safely, while improving their survival rates. The safety of telerehabilitation has also been studied in various works. A recent systematic review studying the adverse effects of cardiac telerehabilitation showed a very low risk of adverse events. The incidence of AEs is estimated at 1 per 23,823 patient hours. There were also no deaths or hospitalizations caused by cardiac telerehabilitation exercises [40,41].

In our findings, most patients showed high adherence of over 90% to telerehabilitation. This result is in line with a meta-analysis of cardiac telerehabilitation in overall cardiovascular disease, reported by Cristo et al. [42]. However, another systematic review from Batalik et al., assessing the use of telerehabilitation in phase two cardiovascular rehabilitation, recorded similar results in training adherence between the two groups. Participants were required to perform vigorous intensity training, which may not be suitable for beginners [39]. Regarding this problem, a hybrid telerehabilitation model could offer a solution to help patients to increase their intensity. 

In addition, telerehabilitation can also bring various advantages, such as minimum transportation, lower costs, ease of customization, possibility of combination with telemonitoring, and finally greater independence [41,43]. A recent systematic review also stated that cardiac telerehabilitation programs can increase physical fitness outcomes and QoL, and reduce costs [43]. However, several challenges are frequently reported, such as a lack of stakeholder acceptability of telehealth, lack of necessary knowledge and skills for e-health, and data protection concerns [44]. Internet access was consistently identified as the most significant barrier to telehealth in developing countries (e.g., the Philippines and Indonesia) across all types of research [44]. In addition, problems of national e-health rules or legislation, lack of governance, and data privacy standards were identified as challenges [44]. Legal and ethical concerns should not be ignored [14]. Telerehabilitation should also adhere to the nursing profession’s ethical and legal standards, but significant gaps remain between advanced technologies and legislation/regulations [45]. The current transmissible outbreak of COVID-19 is a wake-up call. Governments’ political actions should include explicit provisions on multiple fronts for evaluating their telerehabilitation policies and their consequences [46]. To avoid ethical issues, it is emphasized that informed consent should be documented and that medical information must be subject to data protection and confidentiality at all times [45].

The most significant challenge for future studies on telerehabilitation is probably characterizing the barriers and facilitators between nursing, health providers, and people or patients. In addition, a global study is necessary to discover how to implement telehealth in primary care. Additionally, researchers can assess the efficacy of telerehabilitation in various health domains—most notably, in-home nursing for the elderly carries a high risk within the community.

Our study has several limitations. Firstly, some relevant studies were probably overlooked due to the language restriction to English only. Secondly, we lacked access to other databases, including Scopus. Moreover, all the telerehabilitation studies were conducted in developed countries and were limited to certain countries, such as Poland. Several included RCT studies were sub-analyses of one RCT study at the same site, so the sample size was much smaller than expected. Finally, the lack of information during the COVID-19 pandemic substantially limited the possibility of tracing the disease’s genuine management, which can be accomplished only through historical evidence in normal settings.

For future perspectives in this area, the potential integration of artificial intelligence into cardiac rehabilitation is critical. This is a vital topic in mobile health delivery and future development. Artificial intelligence in wearable monitoring and support systems can provide tailored and ambulatory cardiac telerehabilitation. Wearables could accurately detect and identify human physical actions during cardiac telerehabilitation using artificial intelligence algorithms or models, assessing heart function capacity and allowing the longitudinal follow-up of cardiac telerehabilitation [47]. When artificial intelligence is coupled with cardiac telerehabilitation support systems, it may serve to assess observable indications in real time and triage the results, allowing systems to deliver rapid feedback and more tailored recommendations to different grades as preliminary interventions [47]. Furthermore, based on the data, algorithms might automatically refer patients with poor outcomes or difficult scenarios to professionals for additional evaluation [47].

## 5. Conclusions

In conclusion, telerehabilitation can potentially be as safe, effective, and feasible as the cardiac telerehabilitation care delivery model for heart failure patients, especially during transmissible disease outbreaks. Given the benefits and effectiveness of telerehabilitation, patients and health systems are expected to start using telerehabilitation to improve rehabilitation and monitoring services. Health workers should establish protocols that increase patients’ and families’ satisfaction with health service. In addition, the government must also start examining and writing legislation that supports the use of telerehabilitation.

Additional resources should be allocated in the construction of telerehabilitation services, and more high-quality RCTs should be performed to examine the feasibility of telerehabilitation in managing patients with heart failure during transmissible disease outbreaks, particularly COVID-19.

## Figures and Tables

**Table 1:** Characteristics of included studies.

Study	Study Design	Location	Sample			
			Patients’ Diagnoses	Size	Gender (Male%)	Age (Year)
Główczyńska et al. (2021) [25]	RCT	Poland	stable HF with LVEF < 40%	782	*n* = 698	IG: HTCR 65 ± 8.1 UC 63.4 ± 7.9 CG: HTCR 60.6 ± 11.7 UC 61.1 ± 11.2
Piotrowicz et al. (2020) [24]	RCT	Poland	stable HF NYHA class I, II, or III and left LVEF ≤ 40%	859	89.0	IG: 62.2 ±10.9 CG; 62.1 ±10.2
Orzechowski et al. (2021) [28]	RCT	Poland	stable HF NYHA class I, II, or III and left LVEF of 40%	386	89.6	62 ± 11
Piotrowicz et al. (2020) [26]	RCT	Poland	stable HF NYHA class I, II, or III and left LVEF ≤ 40%	425	88.7	62.6 ± 10.8
Piotrowicz et al. (2019) [29]	RCT	Poland	stable HF NYHA class I, II, or III and left LVEF ≤ 40%	850	N/I	N/I
Bernocchi et al. (2018) [22]	RCT	Italy	COPD and HF	112	82.1	70 ± 9
Peng et al. (2018) [23]	RCT	China	a primary diagnosis of chronic HF NYHA class I to III	98	59.2	66.3 ± 10.5
Hwang et al. (2017)-a [30]	RCT	Australia	chronic heart failure with a reduced or preserved ejection fraction	53	75.0	67 ± 12
Hwang et al. (2017)-b [33]	RCT	Australia	HF unspesificy	17	88.0	69 ± 12
Piotrowicz et al. (2016) [31]	RCT	Poland	HF unspesificy	69	*n* = 61	IG 54.3 ± 10.38 CG 60.4 ± 11.9
Piotrowicz et al. (2015)-a [32]	RCT	Poland	stable HFNYHA class II/III with LVEF < 40%	131	85.0	56.4 ± 10.9
Piotrowicz et al. (2015)-b [27]	RCT	Poland	stable HFNYHA class II/III with LVEF < 40%	108	*n* = 95	IG: 54.4 ± 10.9 CG:62.1 ± 12.5

CAD = coronary artery disease; CG = control group; COPD = chronic obstructive pulmonary disease; F = female; LVEF = left ventricular ejection fraction; HF = heart failure; HTCR = hybrid comprehensive telerehabilitation; IG: intervention group; NYHA = New York Heart Association; UC = usual care.

**Table 2:** Models of cardiac telerehabilitation for heart failure.

Type of Telehealth	Model	Input	Process	Output
Hybrid cardiac telerehabilitation (HCT) [24,25,26,27,28,29,31,32,34]	Using a mobile phone for voice communication and TR set. A TR set (Pro Plus Company, Poland) consists of EHO mini device, blood pressure monitoring, and weighing equipment.	First, patients use their phones to answer questions on their current health, including fatigue, dyspnea, symptoms, body mass, and medications taken before starting an exercise session. Second, the EHO mini will record ECG data from three precordial leads and send it to the monitoring center through a mobile phone network. Each patient’s EHO mini device features preprogrammed training sessions (defined exercise duration, breaks, timing of ECG recording). The automated ECG registration is timed to the exercise training. The device indicates what needs to be done via sound and light signals. There are bleeps and light signals from color-emitting LEDs. Bleeps and green diode blinking means that the patient needs to move. “Stop exercise” is indicated by other bleeps and blinking of a red diode. The ECG recording starts automatically coinciding with peak exercise.	Prior to allowing the beginning of the training session, health workers will evaluate data from CIED remote monitoring. Patients are permitted to begin the training session if no contraindications are detected.	Exercise: The monitoring center evaluated the safety, efficacy, and accuracy of the program. By using HR data during exercise and patients’ subjective assessments of the Borg scale, experts could change the training workload or even stop the session if necessary. Psychological support: Telephone interaction was also employed for mental health. Education: All exercise training modalities (the Borg scale and how to run a TR set) were taught. Nutritional counseling, lipid management, smoking cessation, and psychosocial assistance were also included. All outcomes were assessed at baseline and after completing the 9-week program. Patients will be followed-up for a maximum of 24 months.
Home-based telerehabilitation program [22]	Telerehabilitation home-based program (Telerehab-HBP) using smartphone, oximeter, and portable one-lead ECG (Card Guard Scientific Survival Ltd., Rehovot, Israel).	With the cardiologist and pulmonologist directing the program, the nurses made a weekly structured phone call to each patient to collect information about disease status and symptoms, nutrition, lifestyle, and medications. Patients were given a pulse oximeter and a portable one-lead ECG to monitor vital signs in real time. The rehabilitation consisted of light and hard traning. Light training included 15–25 min on a mini-ergometer with no load, 30 min of callisthenic exercises three times a week, and two days of free walking. ‘Hard level’ included 30–45 min of mini-ergometer with total load (0–60 W), 30–40 min of muscle-strengthening exercises with 0.5 kg weights, and pedometer-based walking. Patients might call for any emergency conditions 24 h a day.	Patients were required to report every program’s daily performance and issues during the telephone appointment. The physiotherapist would provide changes in the number and intensity of training sessions every 4 months or when issues arose by assessing the Borg scale at the end of any training session.	General clinical condition: asthenia, muscle pain, and joint pain. Physical activity: duration of exercises and number of steps. Clinical parameters before and after training (blood pressure, heart rate, oxygen saturation, and Borg scale). Education: lifestyle changes and the importance of performing exercise. First follow-up was done 4 months after hospital discharge; 2nd follow-up was done 2 months later. Patients’ satisfaction was measured during the first follow-up. Other outcomes were assessed at both first and second follow-up.
Home-based telehealth exercise training progam [23]	An instant messaging service allows users to communicate online using text, audio, or video. The exercise training program used QQ and Wechat software to communicate and supervise.	Prior to the intervention, an instant messenger (QQ and Wechat) group was created for patients and researchers to communicate. Stage 1 (1–4 weeks) concentrated on endurance workouts, with three 20-min sessions each week. Walking was the most prevalent modality used in the first stage. Patients had 12 20-min exercise sessions three times per week. The second stage (5–8 weeks) included 5 30-min resistance and muscular strengthening sessions. The patients did endurance workouts before moving on to resistance. Walking, jogging, and calisthenics were used to train the muscles. The second stage included 20 30-min workout sessions five days a week. The participants could also contact cardiac nurses through phone or Wechat at any moment. Referral services were also provided if necessary.	During the activity training program, cardiac nurses called or texted to check on patients every week. Questions and responses were followed up with talks regarding the current situation and challenges. Physiotherapists were in charge of monitoring, assessing, and changing the training intensity as needed. The workout prescription prioritized exercise intensity. This intervention measured exercise intensity by target HR, which was determined using the HR reserve method.	Patients’ exercise intensity, evaluated by target HR. Target training HR equals 40–70% of HR reserve + resting HF. Participants were required to complete 3 surveys at discharge (as baseline), 2 months following discharge (post-test 1), and 6 months following discharge (post-test 2).
Home-based telerehabilitation [30,33]	Online video conferencing software	Exercise prescription was adjusted to each participant’s goals and reviewed continually by the treating physiotherapist. Participants could borrow a laptop computer, a mobile broadband device with 3G wireless internet, an automatic sphygmomanometer, a finger pulse oximeter, free weights, and resistance bands. In case of any questions or technical issues, participants could call for technical help by phone. Each participant was instructed to self-monitor and vocally report their blood pressure, heart rate, and oxygen saturation levels. Weight, blood sugar, peripheral edema, and general wellness were also measured if applicable.	The telerehabilitation program was provided to groups of up to four participants in their homes via a synchronous videoconferencing platform.	This 12-week heart failure rehabilitation program included 60 min of exercise at the treating hospital, twice a week. Session length was 40 min, including a 10-min warm-up, followed by a 10-min cool-down. The exercise intensity started at 9 (very mild) and progressively increased to 13 (slightly strenuous). The exercise prescription was adjusted to the participant’s goals and continually reviewed by the treating physiotherapist. Self-management, nutritional and physical activity counseling, psychological therapies, pharmaceuticals, and risk factor management were all covered by multidisciplinary healthcare. Participants were also given home workouts three times per week at the same intensity as the supervised sessions. Assessment were done at baseline, week 12 (1st follow-up), and week 24 (2nd follow up).

ECG = electrocardiography; CIEDs = cardiovascular implantable electronic devices; EHO = ECG recorder; HCT = hybrid comprehensive telerehabilitation; HR = heart rate; TR = telerehabilitation.

**Table 3:** Outcome studies.

Study	Outcome	Intervention	Results		
			Feasibility	Efficacy	Safety
Główczyńska et al. (2021) [25]	Cardiopulmonary exercise capacity (using CPET)	Hybrid cardiac telerehabilitation	N/I	Patients in HCTR group were associated with longer exercise time. The differences in exercise time between HCTR and UC were 12.0 s (95% CI: 15.1–39.1; *p* = 0.666) in DM and 43.1 s (95% CI: 24.0–63.0 s; *p* < 0.001) in non-DM. HCTR group was also associated with lower ventilation at rest compared to UC. The differences were −0.34 L/min (95% CI: 1.60,−0.91 L/min; *p* = 0.892) in DM and 0.83 L/min (95% CI 0.06, 1.73 L/min; *p* = 0.082) in non-DM. In VE/VCO2 slope, a non-significant difference was found: 1.52 (95% CI; 1.55–4.59; *p* = 0.579) for DM vs. − 1.44 (95% CI −3.64–0.77; *p* = 0.336) for non-DM.	Both HCTR and UC are safe in DM and non-DM patients, as evidenced by lack of significant adverse effects experienced by patients.
Piotrowicz et al. (2020) [24]	QoL (SF-36)	Hybrid cardiac telerehabilitation	No patients were exluded or lost to follow-up during 9-week study period.	HCTR significantly improved overall QoL (*p* = 0.009). Greater improvement was observed in HCTR compared to UC group. QoL domain improvement in HCTR group: QoL—physical domain (*p* = 0.0003); QoL—physical functioning (*p* = 0.001); QoL—role functioning related to physical state (*p* = 0.003); QoL—bodily pain (*p* = 0.015).	N/I
Orzechowski et al. (2021) [28]	Safety measured by frequency of cardiac arrhythmias	Hybrid cardiac telerehabilitation	12/425 patients were discontinued for non-medical reasons.	N/I	No patients experienced symptomatic arrhythmia requiring the discontinuation of telerehabilitation. Sinus rhythm was detected in 320 (83%), while persistent atrial fibrillation (AF) was present in 66 (17%) patients. Ventricular and atrial premature beats were the most frequently seen arrhythmias, occurring in 76.4% and 27.7% of patients, respectively. Non-sustained ventricular tachycardia (21 occurrences in 8 patients) and paroxysmal atrial fibrillation (6 episodes in 4 patients) were considered uncommon.
Piotrowicz et al. (2020) [26]	Quality of life (SF-36) and clinical outcome	Hybrid cardiac telerehabilitation	No patients were exluded or lost to follow-up during 9-week study period.	HCTR significantly improved patients’ quality of life (1.58 (95% CI, 0.74–2.42) vs. 0.00 (95% CI, 0.84 −0.84); *p* = 0.008) and peak oxygen consumption (0.95 vs. 0.00 mL/kg/min; *p =* 0.001).	The intervention group did not show improved survival rates (91.9 vs. 92.8 days, with a likelihood of 0.49 (95% CI, 0.46–0.53; *p* = 0.74)), mortality rates (12.5%, vs. 12.4% (HR 1.03 [95% CI, 0.70–1.51])), or hospitalization rates (HR 0.94 (95 percent CI, 0.79–1.13)).
Piotrowicz et al. (2019) [29]	Model (see Table 2)	Hybrid cardiac telerehabilitation	Not applicable	Not applicable	Not applicable
Bernocchi et al. (2018) [22]	Primary: Feasibility and efficacy (6MWD) Secondary: Dyspnea; physical activity; disability; QoL (MRC; PASE: Barthel; MLHFQ)	Home-based telerehabilitation	93% participants performed designed activity at home. Patients’ satisfaction with the program was reportedly very high, with overall mean score 22.3/25.	After 4 months, patients in IG were able to walk further than they did at the beginning: the improvement in 6MWD in IG was 60 (22.2,97.8) m; meanwhile, CG showed no significant improvement (−15 (40.3,9.8)) m. The difference between two groups was significant. IG was associated with significant improvement in the PASE score (*p* = 0.0175), Barthel (*p* = 0.01), and MLHFQ score (*p* = 0.0175) compared to CG at 4 months.	No major side effects were recorded. The IG group was better than CG. Required 113.4 days for the media in IG to reach a hospital or die, compared to 104.7 in the CG (*p* = 0.048, log-rank test). Cumulative hospitalizations happened in 21 patients (IG) and 37 patients (CG).
Peng et al. (2018) [23]	QoL (MLHFQ); 6MWD; resting HR; HADS; LVEF; the NYHA classification	Home-based telehealth exercise training progam	4 patients were lost to follow-up and 3 were omitted from the intervention.	Patients receiving home-based telehealth were associated with significant improvements in QoL, 6MWD, and resting HR. No significant improvements were observed regarding NYHA classification, LVEF, anxiety, and depression at follow-up.	No significant complications or adverse outcomes reported during the program.
Hwang et al. (2017)-a [30]	Primary: 6MWD Secondary: QoL (MLHFQ); patients’ statisfication; attendance rates; adverse events	Home-based telerehabilitation	IG had significantly higher attendance rates than CG, with a mean difference of 6 (95% CI: 2 to 9) sessions.	At Week 12, the IG had a 15 m (95% CI −28 to 59) advantage in the 6MWD (F_(1,6)_ = 1.39; *p* = 0.24). At week 24, IG had a non-significant 2 m (95% CI −36 to 41) advantage compared to CG. Mean within-group QoL difference was 11 (95% CI: −19 to −3).	The number of adverse events was similar between groups. No patients died, had a heart attack, syncope, or fell during the workout period. Both groups reported modest adverse effects, including angina, diaphoresis, and palpitations.
Hwang et al. (2017)-b [33]	Experience and perspective	Home-based telerehabilitation	Participants described telerehabilitation program as easily accessible, safe, and structured.	Participants called for better audio quality and connectivity, as well as computer instruction for individuals who were new to computers. Most participants preferred a combination of face-to-face and online delivery.	N/I
Piotrowicz et al. (2016) [31]	Depression (BDI with cut-off point 20); LF/HF; physical capacity improvement	Hybrid cardiac telerehabilitation with Nordic walking training	All patients in intervention group completed telerehabilitation program.	Depression: IG (8.76 ± 6.73 to 6.70 ± 5.53; *p* = 0.0006) CG (11.57 ± 8.18 9.09 ± 7.34; *p* = 0.0490). Depressive symptoms were substantially reduced in both groups (TG, *p* = 0.0006; CG, *p* = 0.0490). LF/HF: IG (2.06 ± 1.14 to 1.19 ± 0.80; *p* < 0.0001) CG (2.01 ± 1.35 to 2.42 ± 1.39; *p* > 0.05). Between-group differences were significant (*p* = 0.0001). Peak VO2: IG (16.83 ± 3.72 to 19.14 ± 4.20 mL/kg per minute; *p* < 0.0001). Favorable results in CG were not observed. The differences between groups were significant (*p* < 0.0001).	N/I
Piotrowicz et al. (2015)-a [32]	Safety, efficacy, adherence, and acceptance Primary: VO2 peak Secondary: workload duration (t) in 6MWT; QoL (SF-36); safety; adherence and acceptance	Home-based telemonitored Nordic walking in HF patients with CIEDs (i.e., cardiac resynchronization therapy, implantable cardioverter–defibrillator)	All patients completed the program. The adherence was very high: 94.7% patients were adherent, while others were partially adherent. Moreover, 99% participants in IG reported that the device was very easy or easy to use, and 90% had no problems coordinating the exercise.	Nordic walking telerehabilitation training resulted in significant improvement in: VO2 peak (16.1 ± 4.0 vs. 18.4 ± 4.1 mL/kg/min), test duration (471 ± 141 vs. 577 ± 158 s), 6MWD (428 ± 93 vs. 480 ± 87 m), and QoL (79.0 ± 31.3 vs. 70.8 ± 30.3). The improvement differences between IG and CG were significant in ΔVO2 peak (Δ2.0 ± 2.4 vs. Δ−0.2 ± 2.1), Δtest duration (Δ108 ± 108 vs. Δ0.94 ± 109, and Δ6MWT (Δ53.8 ± 63.9 vs. Δ22.0 ± 68.7).	Patients felt safer during telemonitored training than self-exercise without supervision. No deaths, hospitalization, or additional CIED interventions were reported.
Piotrowicz et al. (2015)-b [27]	QoL (SF-36)	Hybrid cardiac telerehabilitation	59/75 patients completed the program.	IG provided similar improvement in overall QoL score to CG group. IG (79.3 ± 25.6 to 70.5 ± 25.4, *p* = 0.007) vs. CG (81.6 ± 27.3 to 69.2 ± 26.4, *p* = 0.004). Significant improvement in IG: physical function (23.2 ± 11.32 to 21.60 ± 9.65, *p* = 0.049) mental health (8.05 ± 3.81 to 7.15 ± 4.03, *p* = 0.012) vitality (8.44 ± 3.36 to 7.25 ± 3.78, *p* = 0.001) Significant improvement in CG: physical function (25.39 ± 10.89 to 23.20 ± 10.71, *p* = 0.044) role limitation caused by physical problems (13.80 ± 7.46 to 11.39 ± 8.43, *p* = 0.034) bodily pain (2.74 ± 2.54 to 2.00 ± 2.07, *p* = 0.011) social function (2.22 ± 1.98 to 1.63 ± 1.54, *p* = 0.005) mental health (7.52 ± 4.51 to 5.89 ± 3.58, *p* = 0.009) vitality (7.94 ± 4.17 to 6.76 ± 3.17, *p* = 0.0197)	N/I

CPET = cardiopulmonary exercise test; ECG = electrocardiography; CI = confidence interval; CIEDs = cardiovascular implantable electronic devices; CG = control group; IG = intervention group; HAD(S) = Hospital Anxiety and Depression Scale; LVEF = left ventricular ejection fraction; MRC = Medical Research Council; MLHFQ = Minnesota Living with Heart Failure Questionnaire; 6MWD = six-minute walking distance; N/I = no information; PASE = Physical Activity Scale for Elderly; LF/HF = low frequency/high frequency; QoL = quality of life.

## Data Availability

Not applicable.

## Supplementary Materials

The following supporting information can be downloaded at: https://www.mdpi.com/article/10.3390/medicina58101321/s1, Supplementary File S1: PRISMA 2020 Main Checklist.

## Appendix A. List of Keywords Used during Literature Search Process

Records that were published in 2012 until 3 March 2022 were considered relevant

Pubmed:
((“telehealth s”[All Fields] OR “telemedicine”[MeSH Terms] OR “telemedicine”[All Fields] OR “telehealth”[All Fields] OR “telenursing”[MeSH Terms] OR “telenursing”[All Fields]) AND (“cardiac rehabilitation”[MeSH Terms] OR (“cardiac”[All Fields] AND “rehabilitation”[All Fields]) OR “cardiac rehabilitation”[All Fields])) AND (“heart failure”[MeSH Terms] OR (“heart”[All Fields] AND “failure”[All Fields]) OR “heart failure”[All Fields] OR ((“congestive”[MeSH Subheading] OR “congestive”[All Field) AND (“heart failure”[MeSH Terms] OR (“heart”[All Fields] AND “failure”[All Fields]) OR “heart failure”[All Fields]))).
ProQuest:
(("telehealth s”[All Fields] OR “telemedicine”[MeSH Terms] OR “telemedicine”[All Fields] OR “telehealth”[All Fields] OR “telenursing”[MeSH Terms] OR “telenursing”[All Fields]) AND (“cardiac rehabilitation”[MeSH Terms] OR (“cardiac”[All Fields] AND “rehabilitation”[All Fields]) OR “cardiac rehabilitation”[All Fields])) AND (“heart failure”[MeSH Terms] OR (“heart”[All Fields] AND “failure”[All Fields]) OR “heart failure”[All Fields] OR ((“congestive”[MeSH Subheading] OR “congestivel”[All Fields]) AND (“heart failure”[MeSH Terms] OR (“heart”[All Fields] AND “failure”[All Fields]) OR “heart failure”[All Fields]))) with applied filters: scholarly journal, full text, peer review, and Publication date 2012–2020.
EBSCO:
(telehealth or telemedicine or telemonitoring or telepractice or telenursing or telecare) AND (cardiac rehabilitation or cardiovascular rehabilitation or cardiac rehab or cardiovascular rehab) AND (heart failure or cardiac failure or CHF or chronic heart failure or congestive heart failure).
